# Alteration of physiological and biochemical properties in leaves and fruits of pomegranate in response to gamma irradiation

**DOI:** 10.1038/s41598-022-08285-y

**Published:** 2022-03-12

**Authors:** Safoora Saadati, Azam Borzouei, Mohammad Reza Rahemi, Behnam Naserian Khiabani

**Affiliations:** Agricultural Research School, Nuclear Science and Technology Research Institute, P. O. Box: 31485/498, Karaj, Iran

**Keywords:** Physiology, Plant breeding

## Abstract

Pomegranate breeding to improve the marketability requires the production of large and high quality fruits. Gamma radiation on pomegranate can be used to generate genetic diversity that allows the breeder to screen the mutants for superior quality and quantity. For this purpose, dormant buds on 1-year-old shoots of pomegranate cultivar "Malase Saveh" were subjected to 36 Gy (Gy) of gamma irradiation from a cobalt (^60^CO) source. Shoot cuttings were taken from the mutated shoots and generate M_1_V_2_. The number of 11 mutants were selected from M_1_V_2_ plants based on their winter survival and disease resistance. After a period of 3–4 years, leaf and fruit samples were harvested from the M_1_V_5_. Results showed that physiological and biochemical parameters of leaves were altered unevenly, some clones showed no alterations from the control, while others revealed considerable differences. Irradiation altered various aspects related to fruit, such as the number and weight of ripe and unripe fruits, number of cracked, sunburn, worm-eaten fruits, and fruit size. In general, mutant clones 5, 8, and 10 had higher fruit sizes and weight of ripe fruits and less number and weight of unripe fruits. The stability of the detected mutants will be evaluated and new commercial field trials using selected materials will be established.

## Introduction

In biological studies, gamma irradiation has been used from low-dose stimulants to high-dose inhibitors^1^. Gamma-ray is widely used as a physical mutagen to induce mutation. To form free radicals, the ionizing radiation gamma ray would interact with atoms or molecules within the cells. Since these radicals can cause substantial damage to DNA or chromosomes within the cell, they may cause a higher incidence of genetic mutation in plants^2^. Gamma-rays have a biological effect by interacting with atoms or molecules in the cell, notably water, to form free radicals^3^. The morphology, anatomy, biochemistry, and physiology of plants are affected differently depending on the irradiation dose by these radicals, which can harm or modify essential components of plant cells^4^. These effects include alterations in plant cell structure and metabolism, such as expansion of thylakoid membranes, changes in photosynthesis, regulation of antioxidant systems, and accumulation of phenolic compounds^3,5^. Antioxidant systems, both enzymatic and non-enzymatic (such as proline and phenolic compounds) play a significant role in scavenging damaging reactive oxygen species (ROS) inside plant cells and defending the plant against numerous stresses, including radiation^6,7^. Excess ROS can react with practically all cell components. As a result of this interaction, chain reactions of free radicals occur, leading to lipid peroxidation of the membrane. Malondialdehyde (MDA), a byproduct of lipid peroxidation, can react with amino acid residues of membrane protein and nucleic acid, lowering membrane stability, and increasing membrane permeability. As a result, cell structure and proper physiological functioning are harmed, resulting in cell aging and organism death because of pathogenic changes. Free radicals can be scavenged by phenolic substances. The existence of conjugated ring structures and hydroxyl groups in phenolic compounds confers antioxidant action, allowing them to operate as reductants, hydrogen donors, and singlet oxygen quenchers^8^. Flavonoids boost the plant's ability to self-protect during irradiation stress (e.g., gamma, UV, and X) by preventing the development of the cellular membrane lipid peroxidation product-MDA and maintaining membrane permeability^9^. In rosemary callus culture, gamma irradiation at 20 Gy increased total phenolic and flavonoid accumulation^10^. Gamma irradiation at 10 Gy enhanced the level of phenolic acid in cinnamon^11^. Similarly, prolonged UV light irradiation of seeds (for 1–6 h) resulted in an increase in lipid peroxidation in wheat sprouts^12^. In mung bean sprouts, the highest total flavonoids content was found in red-blue light with X-ray at a dose of 20 Gy, especially due to the contribution of Kaempferol-rutinoside and rutin^13^.

Changes in physiological characteristics can be aided by gamma irradiation^14^. Many researchers have described physiological effects in a wide spectrum of plants exposed to gamma irradiation^3,5,15,16^. There was clear evidence that numerous environmental conditions, including gamma irradiation, impacted osmolyte production, such as proline, which is involved in protective mechanisms^17^. As the gamma doses rose, the proline content of gamma-irradiated seedlings increased slightly. In irradiated sunflower plants a considerable increase in protein, carbohydrate, and DNA, but a significant decrease in RNA content was discovered^18^.

Proline synthesis was one of the defensive mechanisms in the production of osmolytes, which is necessary for plant growth^19^. Irradiated fenugreek (*Trigonella foenum-graecum* L.) plants had a higher proline content in M1 generation under all gamma doses (25, 50, 100, 200 and 400 Gy) and the greatest amount of proline was obtained at 200 Gy with a significant decrease in the M2 generation under the same dose^20^. Conversely, it has been reported that gamma irradiation in rice var. Tarom (Iranian local rice variety) under drought condition may increase antioxidant levels and eliminate the need for additional proline to deal with the same oxidative reagent problem^21^. Since their direct association with physiological activities including photosynthesis, translocation, and respiration, changes in carbohydrate content are particularly important. The highest levels of soluble sugars were found in gamma irradiation of chrysanthemum cuttings of ‘Emily’ cultivar at a dose of 20 Gy, however, irradiation doses more than 20 Gy had an inhibitory effect^22^.

The *Punicaceae* family includes the pomegranate. It is native to Iran and the Himalayan Mountains in northern India and has been grown and naturalized in the Mediterranean region since antiquity^23^. New pomegranate orchards are being planted in many places of the world due to the increasing awareness of the nutritional value of pomegranate. Although pomegranates are tolerant of a wide range of climatic conditions, the considerable heterogeneity of new growth habitats around the world poses a global challenge to commercially producing high-quality and yielding fruit. Most mutation breeding on pomegranates is focused on cultivars with important traits such as fruit yield, size, juice quality, and good keeping quality mutant seedlings.

Genetic diversity is an essential factor in plant breeding. Using mutation breeding methods, it is possible to create new variations in a short time. The most crucial factor in mutation research is to determine appropriate dose for the species^24^. The regression line between plant growth parameters and radiation dose treatment indicates genetic damage to the plant. To detect the most proper mutant dose, the LD_50_ (the gamma dose that kills 50% of plants) must be determined^25^.

Therefore, in this study, a pre-test was performed to determine the appropriate dose of gamma ray in cutting of pomegranate cultivar "Malase Saveh". The gamma ray doses included zero to 120 Gy. Based on the survival percentages, the LD_50_ of the pomegranates were achieved at 36 Gy^26^. As a result, the goal of this study was to see how gamma irradiation affected several physiological and biochemical properties of leaves and fruits in pomegranate after being exposed to 36 Gy doses of ^60^Co gamma irradiation.

## Results

### Electrolyte leakage (EL)

According to the electrolyte leakage values, a significant difference was detected among the mutant pomegranate clones and control. The highest EL was found in mutant 6 (26.30%), whereas the lowest EL was observed in mutant 7 (17.57%). The control and other mutant pomegranate clones had moderate ion leakage and no significant difference was observed between them in terms of this trait (Table 1). According to Table 4, a significant correlation existed between EL and total soluble carbohydrates (r = − 0.35^*^), MDA (r = 0.44^**^), weight of unripe fruits (r = 0.56^**^).

**Table 1:** Comparison of the means of physiological and biochemical traits in the leaves of new mutant clones of pomegranate c.v. ‘Malase Saveh’.

Mutant clone no	EL (%)	Pro (µmol g^−1^ FW)	TSC (mg g^−1^ FW)	Phenol (mg GAE g^−1^ FW)	DPPH (%)	MDA (nmol g^−1^ FW)	Chlorophyll a (mg g^−1^ FW)	Chlorophyll b (mg g^−1^ FW)	Total chlorophylls (mg g^−1^ FW)	Total carotenoids (mg g^−1^ FW)
Control	22.10 b	11.53 e	16.17 bc	19.92 abc	75.53 a	0.170 i	7.49 a	3.17 bc	10.66 bcd	3.51 bcd
Mut1	21.99 b	25.91 b	19.49 ab	21.01 a	75.03 a	0.200 g	7.02 a	2.17 e	9.19 e	3.21 e
Mut2	23.45 b	21.42 c	20.37 a	14.25 f	75.52 a	0.227 b	7.64 a	4.01 a	11.65 ab	3.68 b
Mut3	21.35 b	13.39 e	19.31 ab	16.32 e	74.65 a	0.227 b	7.28 a	2.31 de	9.59 de	3.48 cd
Mut4	21.99 b	23.45 bc	16.41 abc	20.71 ab	75.03 a	0.183 h	7.48 a	3.28 bc	10.76 bc	3.53 bcd
Mut5	20.08 bc	33.28 a	16.45 abc	18.56 cd	74.90 a	0.157 j	7.52 a	3.19 bc	10.72 bcd	3.47 cd
Mut6	26.30 a	13.21 e	10.00 d	19.29 bc	74.07 a	0.260 a	7.49 a	2.68 cde	10.17 cde	3.65 bcd
Mut7	17.57 c	11.29 e	13.51 cd	17.35 de	73.49 a	0.207 f	7.46 a	3.09 bc	10.55 bcd	3.46 d
Mut8	23.59 b	13.70 e	16.72 abc	17.68 de	72.59 a	0.220 d	7.50 a	2.86 cd	10.36 cd	3.66 bc
Mut9	22.03 b	11.06 e	13.18 cd	19.60 abc	72.97 a	0.210 e	7.55 a	3.57 ab	11.12 bc	3.49 bcd
Mut10	20.93 bc	16.76 d	16.94 abc	20.78 ab	72.20 a	0.223 c	7.51 a	3.05 bc	10.57 bcd	3.52 bcd
Mut11	22.44 b	24.19 bc	16.38 abc	21.19 a	71.62 a	0.223 c	8.40 a	4.07 a	12.46 a	3.90 a

Means in each column followed by the same lower case letters were not significantly different by LSD at p ≤ 0.05.

*EL* electrolyte leakage, *Pro* proline, *TSC* total soluble carbohydrates, *Phenol* total phenolics, *DPPH* DPPH scavenging capacity, *MDA* malondialdehyde, *Chlorophyll a* chlorophyll a, *Chlorophyll b* chlorophyll b, *Total chlorophylls* total chlorophylls, *Total carotenoids* total carotenoids.

### Proline

As shown in Table 1, results demonstrated that the tested mutants revealed leaf proline concentrations on a limited range from 11.06 to 33.28 µmol g^−1^ FW. Mutant 5 (33.28 µmol g^−1^ FW) showed the highest proline content, however, the lowest proline content was observed in mutants 9, 7, 6, 3, and 8 (11.06, 11.29, 13.21, 13.39, 13.70 µmol g^−1^ FW), which were not significantly different with the control (Table 2). Significant correlations were found between proline content and soluble carbohydrates (r = 0.37^*^), MDA (r = − 0.36^*^), and number of worm-eaten fruits (r = − 0.34^*^) (Table 4).

**Table 2:** Comparison of the means of number and weight of ripe and unripe fruits, number of cracked, sunburn, and worm-eaten fruits of new mutant clones of pomegranate c.v. ‘Malase Saveh’.

Mutant clone no	Number of ripe fruits/tree	Weight of ripe fruits (kg/tree)	Number of unripe fruits/tree	Weight of unripe fruits (kg/tree)	Number of unripe/ripe fruits	Number of cracked fruits/tree	Number of sunburn fruits/tree	Number of worm-eaten fruits/ tree
Control	15.67 b	2.86 e	23.67 b	1.42 c	1.52 cd	17.00 a	0.33 f	0.33 ef
Mut1	6.66 g	1.96 h	8.33 e	0.38 f	1.25 cd	1.33 e	4.33 e	1.00 de
Mut2	7.33 fg	2.33 g	5.67 f	0.60 e	0.79 cd	5.67 cd	4.67 e	1.67 d
Mut3	3.66 h	3.14 d	45.00 a	1.72 b	12.96 a	5.33 d	7.67 d	0.00 f
Mut4	4.33 h	2.35 g	15.67 c	1.72 b	3.94 b	7.67 b	9.67 c	2.67 c
Mut5	13.33 c	4.05 b	14.67 c	0.59 e	1.10 cd	16.33 a	7.67 d	0.00 f
Mut6	10.00 de	3.25 d	24.00 b	2.15 a	2.40 bc	5.00 d	12.67 b	2.67 c
Mut7	12.00 cd	3.51 c	1.00 h	0.13 h	0.08 d	5.00 d	9.67 c	4.67 a
Mut8	20.00 a	5.05 a	10.33 d	0.64 e	0.52 d	6.67 bc	14.00 a	3.67b
Mut9	9.00 ef	2.56 f	6.67 f	0.38 f	0.74 cd	5.67 cd	9.67 c	0.67 ef
Mut10	9.00 ef	4.06 b	10.67 d	0.84 d	1.19 cd	7.67 b	10.67 c	4.67 a
Mut11	10.33 de	2.37 g	2.67 g	0.26 g	0.25 d	5.67 cd	10.67 c	0.00 f

Means in each column followed by the same lower case letters were not significantly different by LSD at p ≤ 0.05.

### Total soluble carbohydrate (TSC)

According to Table 1, significant differences were observed in the leaf soluble carbohydrate contents among mutant clones and control pomegranates. Mutant clone 2 was revealed to have the highest contents of leaf soluble carbohydrate (20.37 mg g^−1^ FW, respectively), which was significantly more than the control. The lowest leaf soluble carbohydrate content was found in mutant 6 (10.00 mg g^−1^ FW). However, no significant difference was observed between other mutant clones with control in terms of leaf soluble carbohydrates. According to Table 4, a significant correlation existed between leaf soluble carbohydrate and electrolyte leakage (r = − 0.35^*^) proline (r = 0.37^*^), number of sunburn fruits (r = − 0.41^*^), and TSS/TA (r = 0.36^*^).

### Total phenolics

The studied mutants differed significantly in terms of the total phenolic contents. Table 1 shows that the lowest total phenolic contents were seen in mutant 2 (14.25 mg GAE g^−1^ FW) and the highest was found in mutant 11 (21.19 mg GAE g^−1^ FW). However, no significant difference was observed between mutants 1, 4, 5, 6, 9, 10, and 11 with control in terms of total phenolics. No significant correlation was observed between total phenolics and measured traits (Table 4).

### The capacity of DPPH scavenging

The results of the study illustrated that no significant difference was observed between mutant clones and control in terms of 2,2-diphenyl-1-picrylhydrazyl (DPPH) scavenging capacity (Table 1). As revealed by Table 4, there existed a negative correlation between DPPH scavenging capacity and Chlorophyll a, b, and T, Total carotenoids, and MDA (r = − 0.35^*^, − 0.39^*^, − 0.40^*^, − 0.41^*^ and − 0.37 ^*^), respectively.

### Malondialdehyde (MDA)

According to Table 1, mutants significantly differed in lipid peroxidation, measured as MDA content. MDA content generally ended up the least in mutant 5 (0.157 nmol g^−1^ FW), which was significantly less than the control (0.170 nmol g^−1^ FW), whereas the highest MDA content was found in mutant 6 (0.260 nmol g^−1^ FW). As shown in Table 4, a significant and negative correlation was detected between MDA content and the number of cracked fruits, proline, and DPPH scavenging capacity (r = − 0.60^**^, − 0.36^*^, and − 0.37^*^, respectively). Moreover, MDA content was positively and significantly correlated with electrolyte leakage and total carotenoids (r = 0.44^**^, and 0.37^*^).

### Chlorophylls and total carotenoids

According to the chlorophyll b, total chlorophylls, and total carotenoids values, a significant difference was detected among the mutant clones and control pomegranates. mutant clones 11 and 2 showed the highest content of chlorophyll b (4.07 and 4.01 mg g^−1^ FW, respectively); while mutant clones 1 and 3 revealed the least chlorophyll b (2.17 and 2.31 mg g^−1^ FW, respectively). No significant difference was detected between control and other mutant clones in chlorophyll b contents (Table 1). Chlorophyll b contents were in good correlation with the chlorophyll a, total chlorophylls, and total carotenoids values (r = 0.73^**^, 0.96^**^, and 0.73^**^, respectively). In addition, there was a significant negative correlation between chlorophyll b and the number of unripe fruits and the number of unripe/ripe fruits (r = − 0.47^**^ and − 0.40^*^, respectively) (Table 4).

The highest and the lowest total chlorophylls contents were observed in mutants 11 and 1 (12.46 and 9.19 mg g^−1^ FW), respectively. While there was no significant difference between the control and other clone mutants in total chlorophylls contents (Table 1). A positive correlation was also detected to exist between total chlorophylls and chlorophyll a, chlorophyll b, and total carotenoids values (r = 0.90^**^, 0.96^**^, and 0.84^**^, respectively) (Table 4). As shown in Table 4, a significant and negative correlation was also detected between total chlorophylls content and DPPH scavenging capacity, the number of unripe fruits, and the number of unripe/ripe fruits (r = − 0.40^*^, − 0.41^*^ and − 0.34^*^, respectively).

As revealed in Table 1, total carotenoid values of the leaf tissue varied among mutant clones. mutant 11 had the highest level of total carotenoids (3.90 mg g^−1^ FW), whereas mutant 1 had the lowest total carotenoids (3.21 mg g^−1^ FW). Other mutant clones had statistically similar total carotenoids to the control. there were good correlations between total carotenoids and chlorophyll a, chlorophyll b, and total chlorophylls values (r = 0.87^**^, 0.73^**^, and 0.84^**^, respectively). Moreover, a significant correlation was also detected between total carotenoid content and DPPH scavenging capacity and MDA (r = − 0.41^*^ and 0.37^*^, respectively) (Table 4).

### Number and weight of ripe fruits

The results of the study illustrated that the mutants varied in terms of the number of ripe fruit per tree. The highest number of ripe fruit was detected in mutant clone 8 (20), which was significantly more than the control (15.67). Other mutant clones had fewer ripe fruit than control. On the other hand, the lowest number of ripe fruit was found in mutant clones 3 and 4 (3.66 and 4.33) (Table 2). As revealed by Table 4, there existed a significant relationship between the number of ripe fruit and weight of ripe fruits, the number of unripe/ripe fruits and the number of cracked fruits (r = 0.65^**^, − 0.54^**^, and 0.45^**^, respectively).

The results of the study illustrated that the mutant clones varied in weight of ripe fruits. Mutant clones 1, 2, and 9 had statistically less weight of ripe fruits than the control. While other mutants had more fruit weight than the control and the highest weight of ripe fruits was detected in mutant 8 (Table 2). As shown in Table 4, a significant and positive correlation was also detected between the weight of ripe fruits and the number of sunburn fruits, and the number of worm-eaten fruits (r = 0.51^**^ and 0.47^**^, respectively).

### Number and weight of unripe fruits

The highest number of unripe fruits was observed in mutant 3 (45), followed by mutant 6 and control (24 and 23.67, respectively). There was no statistically significant difference between mutant 6 and control in this trait. Other mutant clones had fewer unripe fruits than the control (Table 2). As shown in Table 4, the number of unripe fruits had a significant negative correlation with Chlorophyll b and Total chlorophylls (r = − 0.47^**^ and − 0.41^*^, respectively), and a significant positive correlation with the weight of unripe fruits and number of unripe/ripe fruits (r = 0.80^**^ and 0.85^**^, respectively).

Mutants 6, 3, and 4 had significantly more weight of unripe fruits than the control, while other mutants showed less weight of unripe fruits than the control (Table 2). A positive and significant correlation was observed between the weight of unripe fruits and electrolyte leakage, the number of unripe fruits, and the number of unripe/ripe fruits (r = 0.58^**^, 0.56^**^, and 0.80^**^, respectively) (Table 4).

### Number of unripe/ripe fruits

Mutant clones 3 and 4 had a higher number of unripe/ripe fruits than control, while other mutant clones did not differ significantly from control in this trait (Table 2). As revealed by Table 4, there was a significant negative correlation between the number of unripe/ripe fruits and Chlorophyll b, Total chlorophylls, and the number of ripe fruits (r = − 0.40^*^, − 0.34^*^, and − 0.54^**^, respectively). In addition, a positive and significant correlation was observed between the number of unripe/ripe fruits and umber of unripe fruits, and weight of unripe fruits (r = 0.85^**^ and 0.58^**^, respectively).

### Number of cracked, sunburn, and worm-eaten fruits

Mutant clone 5 and control had the same number of cracked fruits, which was significantly higher than other mutant clones (Table 2). As shown in Table 4, a significant correlation was also detected between the number of cracked fruits and MDA, the number of ripe fruits, and the number of sunburn fruits (r = − 0.60^**^, 0.45^**^, and − 0.37^*^, respectively).

The highest number of sunburn fruits was related to mutant 8, although all mutant clones had significantly more sunburn fruits than the control (Table 2). As shown in Table 4, the number of sunburn fruits had a significant negative correlation with total soluble carbohydrates and number of cracked fruits (r = − 0.41^*^ and − 0.37^*^, respectively), and a significant positive correlation with the weight of ripe fruits and the number of worm-eaten fruits (r = 0.51^**^ and 0.50^**^, respectively).

The lowest number of worm-eaten fruits was found in mutant clones 3 and 11 and the highest was found in mutant clones 10. However, no significant difference was observed between mutant clones 1, 3, 5, 9, and 11 with the control in terms of this trait (Table 2). The number of worm-eaten fruits was significantly correlated with proline, the weight of ripe fruits, and the number of sunburn fruits (r = − 0.34^*^, 0.47^**^, and 0.50^**^, respectively) (Table 4).

### Volume, diameter and length of fruits

The highest and lowest fruit volumes were observed in mutant clones 7 and control, respectively, although all mutant clones had higher fruit volumes than control (Table 3). The fruit volume was significantly correlated with fruit diameter, fruit length, fruit shape index (r = 0.83^**^, 0.85^**^, and − 0.35^*^, respectively) (Table 4).

**Table 3:** Comparison of the means of physical and sensory attributes of the fruit of new mutant clones of pomegranate c.v. ‘Malase Saveh’.

Mutant clone no	Fruit volume (mm^3^)	Fruit diameter (mm)	Fruit length (mm)	Fruit shape index	TSS (ºBrix)	TSS/TA
Control	143.3 e	155.2 f	168.1 f	1.08 a	16.17 a	12.34 a
Mut1	336.7 b	180.7 bc	192.1 bc	1.06 a	16.37 a	11.32 a
Mut2	240.0 d	173.2 bcde	181.0 de	1.05 a	15.27 a	11.39 a
Mut3	326.7 b	182.0 ab	192.7 ab	1.06 a	15.97 a	11.92 a
Mut4	250.0 cd	164.7 ef	176.0 ef	1.07 a	15.57 a	11.02 a
Mut5	286.7 bcd	174.7 bcde	185.1 bcde	1.06 a	16.77 a	11.92 a
Mut6	230.0 d	168.6 de	179.4 e	1.06 a	15.83 a	11.23 a
Mut7	431.7 a	192.6 a	202.9 a	1.05 a	15.37 a	11.03 a
Mut8	316.7 b	178.6 bcd	190.6 bcd	1.07 a	16.40 a	12.08 a
Mut9	310.0 bc	174.5 bcde	184.9 bcde	1.06 a	15.73 a	11.74 a
Mut10	286.7 bcd	172.5 bcde	182.9 bcde	1.06 a	16.63 a	11.85 a
Mut11	286.7 bcd	171.1 cde	182.1 cde	1.06 a	16.00 a	12.03 a

Means in each column followed by the same lower case letters were not significantly different by LSD at p ≤ 0.05.

**Table 4:** Pearson correlation coefficients between some physiological and biochemical traits of leaves and fruit quantitative and qualitative parameters in the mutant clones of ‘Malase Saveh’ pomegranates.

	1	2	3	4	5	6	7	8	9	10	11	12	13	14	15	16	17	18	19	20	21	22	23	24
1	1																							
2	ns	1																						
3	− 0.35*	0.37*	1																					
4	ns	ns	ns	1																				
5	ns	ns	ns	ns	1																			
6	0.44**	− 0.36*	ns	ns	− 0.37*	1																		
7	ns	ns	ns	ns	− 0.35*	ns	1																	
8	ns	ns	ns	ns	− 0.39*	ns	0.73**	1																
9	ns	ns	ns	ns	− 0.40*	ns	0.90**	0.96**	1															
10	ns	ns	ns	ns	− 0.41*	0.37*	0.87**	0.73**	0.84**	1														
11	ns	ns	ns	ns	ns	ns	ns	ns	ns	ns	1													
12	ns	ns	ns	ns	ns	ns	ns	ns	ns	ns	0.65**	1												
13	ns	ns	ns	ns	ns	ns	ns	− 0.47**	− 0.41*	ns	ns	ns	1											
14	0.56**	ns	ns	ns	ns	ns	ns	ns	ns	ns	ns	ns	0.80**	1										
15	ns	ns	ns	ns	ns	ns	ns	− 0.40*	− 0.34*	ns	− 0.54**	ns	0.85**	0.58**	1									
16	ns	ns	ns	ns	ns	− 0.60**	ns	ns	ns	ns	0.45**	ns	ns	ns	ns	1								
17	ns	ns	− 0.41*	ns	ns	ns	ns	ns	ns	ns	ns	0.51**	ns	ns	ns	− 0.37*	1							
18	ns	− 0.34*	ns	ns	ns	ns	ns	ns	ns	ns	ns	0.47**	ns	ns	ns	ns	0.50**	1						
19	ns	ns	ns	ns	ns	ns	ns	ns	ns	ns	ns	ns	ns	ns	ns	ns	ns	ns	1					
20	ns	ns	ns	ns	ns	ns	ns	ns	ns	ns	ns	ns	ns	ns	ns	ns	ns	ns	0.83**	1				
21	ns	ns	ns	ns	ns	ns	ns	ns	ns	ns	ns	ns	ns	ns	ns	ns	ns	ns	0.85**	0.99**	1			
22	ns	ns	ns	ns	ns	ns	ns	ns	ns	ns	ns	ns	ns	ns	ns	ns	ns	ns	− 0.35*	− 0.56**	− 0.41*	1		
23	ns	ns	ns	ns	ns	ns	ns	ns	ns	ns	ns	ns	ns	ns	ns	ns	ns	ns	ns	ns	ns	ns	1	
24	ns	ns	0.36*	ns	ns	ns	ns	ns	ns	ns	ns	ns	ns	ns	ns	ns	ns	ns	ns	ns	ns	ns	0.79**	1

1: electrolyte leakage; 2: proline; 3: total soluble carbohydrates; 4: total phenolics; 5: DPPH scavenging capacity; 6: malondialdehyde; 7: chlorophyll a; 8: chlorophyll b; 9: total chlorophylls; 10: total carotenoids; 11: number of ripe fruits; 12: weight of ripe fruits; 13: number of unripe fruits; 14: weight of unripe fruits; 15: number of unripe/ripe fruits; 16: number of cracked fruits; 17: number of sunburn fruits; 18: number of worm-eaten fruits; 19: fruit volume; 20: fruit diameter; 21: fruit length; 22: fruit shape index; 23: TSS; 24: TSS/TA.

^ns^, *, **Not significant and significant at p ≤ 0.05 or p ≤ 0.01, respectively.

The lowest diameter and length of fruit were related to mutant 4, which was not significantly different from the control. Other mutant clones had significantly larger fruit diameters than control (Table 3). As shown in Table 4, a significant correlation was observed between fruit diameter and fruit volume, fruit length, and fruit shape index (r = 0.83^**^, 0.99^**^, and − 0.56^**^, respectively). Moreover, the fruit length was significantly correlated with the fruit shape index (r = − 0.41^*^).

### Total soluble solids (TSS), and titratable acidity (TA)

No significant difference was observed between mutant clones and control in terms of TSS and TSS/TA (Table 3). As shown in Table 4, the TSS/TA was significantly correlated with leaf soluble carbohydrates and TSS (r = 0.36* and 0.79**, respectively).

## Discussion

Plant mutation breeding methods employ gamma irradiation to improve the qualitative and quantitative characteristics of numerous tree species^27–30^. Because of their availability and penetrating capacity, gamma rays are a more cost-efficient and effective instrument than other types of ionizing radiation. In general, free radicals produced by ionization can impair plants' physiological and biochemical characteristics^31,32^. In other situations, however, low-level ionizing radiation is known to have a favorable influence on plant development, a phenomenon known as hormesis^33^. ROS are produced as a result of oxidative damage caused by a variety of biotic and abiotic stresses^34^. ROS, which is a potent oxidative stress agent that damages lipids, proteins, and DNA within plant cells, can be induced by gamma radiation^35–37^. Increased production of ROS causes lipid peroxidation and electrolyte leakage, lowering membrane stability^38^. Irradiation of pomegranate dormant buds showed distinct physiological and biochemical reactions, according to our findings. For example, mutant clone 6 had the highest levels of EL, MDA, and the weight of unripe fruits, which may be due to the induction of free radicals by gamma irradiation. Mutant clone 7 had lower levels of EL, MDA, and the weight of unripe fruits (Tables 1, 2). Furthermore, there was a positive association between EL, MDA, and the weight of unripe fruits (Table 4). In other words, increased membrane lipid peroxidation (MDA production) increases electrolyte leakage, declines membrane stability, and boosts membrane permeability. As a result, cell structure and normal physiological functioning are damaged and the transfer of photosynthetic compounds across the plasma membrane from source (leaf) to sink (fruit) is disrupted, slowing fruit ripening and increasing unripe fruit.

Plants have evolved several defensive mechanisms to counteract the effects of ROS in cellular compartments to avoid oxidative damage. Proline synthesis was one of the defensive processes in the synthesis of osmolytes, which is necessary for plant growth^14^. This study indicated a substantial negative association between proline concentration and MDA, as well as the number of worm-eaten fruits (Table 4). For example, mutant clone 5 had more proline and fewer worm-eaten fruits, and less MDA, indicating a protective role of proline against ROS and membrane protection.

Changes in carbohydrate content are especially important because they are directly related to physiological processes such as photosynthesis, translocation, and respiration^22^. As seen in Table 1, leaf soluble carbohydrates in mutant clone 2 were higher than the control, but in mutant clone 6 was lower than the control. Soluble sugars in chrysanthemum cuttings were in the highest contents in the low dose gamma irradiation. However, high-dose gamma radiation resulted in an inhibitory effect^22^. Several studies have been conducted on rice to investigate gamma-induced changes in carbohydrate composition and molecular arrangements associated with the functional properties of rice varieties^39–41^. Moreover, a significant correlation was observed between leaf soluble carbohydrates and the number of sunburn fruits (r = − 0.41^*^), and TSS/TA (r = 0.36^*^). Our findings support a previous study that found that soluble carbohydrate levels were higher in sunburned apples^42^. In another study, sunburned apple fruits with mild, moderate, and severe symptoms had significantly higher soluble sugars contents than asymptomatic tissues^43^. In sun-exposed tissues, carbohydrate accumulation may also be a consequence of a lack of metabolism of these molecules due to environmental stress associated with the development of sunburn^44^. The wide range of ROS produced under these conditions can act as signal messengers that induce lignin and synthesize suberin in sun-exposed tissues to strengthen cell walls^45^.

Irradiation caused alterations in total phenolic content in the leaves of pomegranate mutants, according to the findings. Overall, gamma irradiation causes metabolic changes in the leaves and appears to affect phenolic compound formation. In line with our findings, it has been reported that irradiation altered the overall phenolic and flavonoid content of citrus mutant fruit peel and pulp, as well as leaves^46^. Gamma irradiation using 10 kGy also enhanced the phenolic acid content of cloves and cinnamon, but did not change the phenolic acid content of nutmeg^11,47^. The increased total phenolic content can be explained by the release of these compounds from glycosidic form and the destruction of larger compounds into smaller ones under the action of gamma irradiation^48^. Conversely, a decrease in total phenolic compounds in rosemary was observed after irradiation at a dose of 10–30 kg, compared with the control^49^. Differences in the effect of radiation on total phenol content may be due to gamma radiation dose, sample state (solid or dry), type of extraction solvent, phenol content composition, extraction process, temperature, geographical and environmental conditions, plant type^50^.

The chlorophyll content of the irradiated plantlets revealed an uneven distribution in this investigation. Mutant clone 1 had lower chlorophyll b, total chlorophylls, and total carotenoid content, followed by while mutant clone 11 had higher as compared to control. Moreover, the chlorophyll a was not affected by irradiation. Chlorophyll is an important pigment to plants because the light energy it absorbs is used to produce biomass during photosynthesis. The percentage increase in chlorophyll content above the control may be attributed to the fact that the low dose caused an improvement in the photosynthetic capabilities of irradiated plants while high levels of gamma irradiation reduce photosynthesis by disrupting chlorophyll biosynthesis or breakdown, resulting in a reduction in photosynthetic capability^51^. This could also be due to the reduction of the organized pattern of grana and stroma of the thylakoids by the treatments^52^. In pervious study, the application of high doses of gamma rays on lulo plants (*Solanum quitoense* Lam.) caused leaf chlorosis, necrosis of petiole base areas, leaf fall, stunting growth, and death^53^. Total chlorophyll content at high doses 40 and 50 Gy in the *Citrus sinensis* plantlet demonstrated a drastic reduction of 59.1 and 76.9%, respectively^30^. At doses ranging from 2 to 30 Gy, the level of photosynthetic pigments (chlorophyll a, chlorophyll b, and carotenoids) increased in lettuce, but it fell at higher levels (up to 70 Gy)^36^. The total chlorophyll content of red pepper (16 Gy) and lupine (20 Gy) increased significantly after irradiation^16,54^. Furthermore, chlorophyll is virtually unaffected by low levels of gamma irradiation^15^. It was previously observed that gamma irradiation reduced the amount of chlorophyll b more than chlorophyll a 48 in a previous investigation^55^. Gamma irradiation caused an increase in chlorophyll a, b, and total chlorophyll levels in *Paulownia tomentosa* plants^56^.

In our study, Mutant clones 5, 7, 8, and 10 had more weight of ripe fruits and on the contrary, showed less number and weight of unripe fruits compared with control. There are also reports of an increase in the yield of crops treated with mutagens^57–61^. In pervious study on eight cultivars of low-seeded irradiated mandarin, it was observed that the average weight of fruit in one of them was significantly higher than the varieties of non-irradiated trees^62^. Gamma irradiation at a dose of 10 Gy increased the growth parameters in bananas compared to the control group^63^. The positive effects of gamma radiation may be due to the role of mutagens in stimulating the activity of enzymes and hormones responsible for growth and yield^64,65^. Conversely, a 40% decline in barley yield with 30 krads of gamma irradiations. The adverse effects of gamma rays may have caused sterility in the plants, resulting in a decrease in production^66^.

In our study, all studied mutant clones had significantly higher fruit length, diameter, and volume compared to the control. In line with our study, gamma irradiation of pollen grains at a dose of 200–300 Gy increased citrus fruit growth in terms of length, diameter, and volume^29^. Gamma irradiation of bud woods of “Moncada” mandarin altered qualitative characteristics of the fruit including length, diameter in some of the clones assayed^67^. According to our results, all mutant clones except mutant clone 5 had lower fruit cracking rates than control. Similar to our observations, in sweet cherry mutants was observed a decrease in fruit cracking rate^68^.

## Conclusions

Irradiating the buds of plants is an effective method for improving cultivars, producing high-yielding cultivars, and increasing fruit sizes. In this paper, we characterized 11 clones obtained from irradiated bud wood of the pomegranate c.v. Malase Saveh, assessing the physiological and biochemical traits of leaves and some fruit quantitative and qualitative parameters. Mutant clone 11 had significantly more proline, chlorophyll b, total chlorophylls, and total carotenoids than the control and showed less number and weight of unripe fruit and worm-eaten fruit compared to the control. All of the irradiation clones tested had greater fruit length, diameter, and volume than the control, as well as less fruit cracking. Most clones differed noticeably in numerous factors relating to fruit yield after exposure to irradiation, not just in terms of quality. Given the above results, three clones (5, 8, and 10), which demonstrate excellent traits regarding fruit quantity and quality, including higher fruit size and weight of ripe fruits, and lower number and weight of unripe fruits, are the subject of further studies.

## Materials and methods

**Figure 1:**
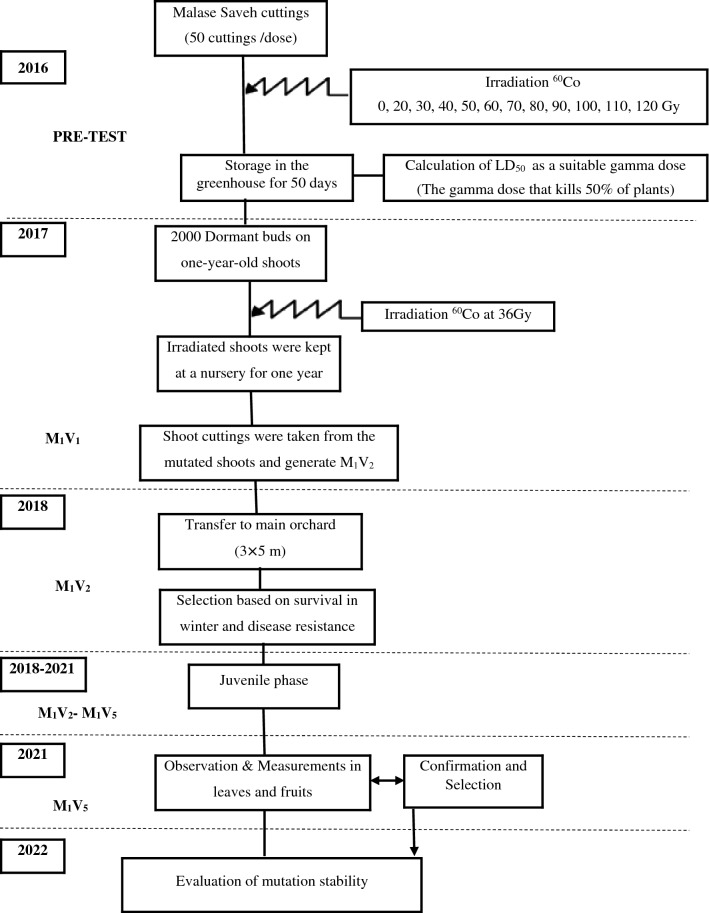
A schematic diagram illustrating the process of mutant production in pomegranate.

This study was conducted at the Agriculture Research School, Nuclear Science and Technology Research Institute, Karaj, Iran. In the pre-test, buds on 1-year-old pomegranate shoots were exposed to gamma-ray doses including 0, 20, 30, 40, 50, 60, 70, 80, 90, 100, 110 and 120 Gy (Gy). Linear regression between cuttings survival and gamma ray doses was estimated. According to the LD_50_ radiation dose, 36 Gy was determined as the appropriate gamma dose rate during the experiment^26^. In next year, 2000 dormant buds on 1-year-old pomegranate shoots were subjected to gamma irradiation at a dose of 36 Gy from a cobalt (^60^CO) source. After that, irradiated shoots were kept at a nursery for about a year, and these mutant plants were used for vegetative propagation and production of second mutant vegetative generation (M_1_V_2_) plants. In the winter of the following year, mutant clones M_1_V_2_ were relocated to the main garden in Saveh in Markazi province and planted in east–west rows in a sandy loam soil using a randomized complete block design with three replications. All of the growing and horticultural practices such as irrigation, fertilization, and disease control were the same for both pomegranate control and mutant clones. Plants from M_1_V_2_ were selected based on their survival in winter and disease resistance. There were 11 mutants selected from M_1_V_2_. After a growth period of 3–4 years, all the trees entered the flowering stage. One-year-old shoots were harvested from the M_1_V_5_ in May 2021 and stored on ice until utilizing them in a lab for analyzing physiologic and biochemical features. In November 2021, the fruit from these plants analyzed for number of fruit produced and the quality. The correlation between leaf physiological and biochemical traits and fruit yield was then investigated. Time and methods scheme of experiment are shown in Fig. 1.

### Physiological and biochemical assays

#### Electrolyte leakage (EL)

Electrolyte leakage of the leaves was prepared as described previously with modification^69^. Five leaf discs, each with a diameter of one centimeter, were cut from each replication. Samples were washed with deionized water, placed in test tubes containing 10 mL distilled water, and incubated at room temperature for 24 h on a shaker. An electrical conductivity meter (CC-501, Elmetron, Zabrze, Poland) was used to determine the initial electrical conductivity (EC_1_) of each solution. After that, the sample was placed in a boiling water bath for an hour to kill all of the tissues and allow for maximum ion leakage. After cooling the sample to room temperature, the EL was calculated once more (EC_2_). Electrolyte leakage was calculated using the formula EC_1_/EC_2_ and then expressed as a percentage.

#### Proline

Proline content was determined as described previously^70^. After homogenizing the leaf tissue in liquid nitrogen, 0.5 g of the sample (10 leaves from 3 trees of each replicate) was combined with a 10 mL aliquot of 3% sulfosalicylic acid in capped glass tubes, and then centrifuged at 5000*g* for 10 min. Two milliliters of the clear supernatant were combined with two milliliters of freshly made acid ninhydrin reagent and two milliliters of glacial acetic acid and then heated for one hour on a water bath. After the mixture had cooled, 4 mL toluene was added and rapidly agitated for 20 s. Toluene was used as a blank and was used to detect free proline spectrophotometrically at 520 nm. Free proline was calculated through a calibration curve and expressed as μmol g^−1^ FW.

#### Total soluble carbohydrate (TSC)

The total soluble carbohydrate content of leaf tissue was determined using the anthrone technique^71^. Briefly, 0.5 g of plant tissue (10 leaves from 3 trees of each replicate) was crushed in liquid nitrogen, then extracted three times with ethanol at 20 °C and centrifuged at 1500*g* for 15 min. In 100 mL of ethanolic extract, three milliliters of anthrone reagent (150 mg anthrone in 100 mL of 72% sulfuric acid) were added. The reaction mixture was heated for 10 min in a boiling water bath before being quickly cooled on ice. The absorbance of the cooled sample was then measured at 625 nm using a spectrophotometer (UV 160A, Shimadzu Corp., Kyoto, Japan). The results were derived using a glucose standard curve and expressed in milligrams per gram of FW.

#### Total phenolics

Total phenolics were measured as described previously using 1 g of leaf sample (10 leaves from 3 trees of each replicate) extracted in 10 mL of methanol^72^. In a test tube, the methanolic extract (125 μL) was combined with 375 μL of distilled water, then 2.5 mL of 10% Folin Ciocalteu reagent was added, and the mixture was allowed to stand for 6 min. After that, 2 mL 7.5% Na_2_CO_3_ was added. Each sample was incubated for 90 min at room temperature in the dark before being measured at 760 nm for absorbance. The phenolic content of the extract was calculated using an equation derived from the standard gallic acid (GAE) curve and reported as mg GAE g^−1^ FW.

#### The capacity of DPPH scavenging

The diphenyl-2-picrylhydrazyl (DPPH) radical technique was used to determine the free radical scavenging activity of pomegranate extracts, with some modifications^73^. As a result, 10 mL of methanol was used to extract 1 g of the leaf samples (10 leaves from 3 trees of each replicate). After that, 50 μL of methanolic extract were added to 950 μL of 0.1 mM DPPH radical and vortexed; the mixture was then kept at room temperature for 30 min before the absorbance at 517 nm was measured. The following is how the scavenging effect was achieved:

$${\text{DPPH scavenging}}\,\% = \left[ {\left( {{\text{A}}_{{{517}}}\,{\text{control}} - {\text{A}}_{{{517}}}\,{\text{sample}}} \right)/{\text{A}}_{{{517}}}\,{\text{control}}} \right] \times {1}00$$

#### Malondialdehyde (MDA)

The MDA content was determined using spectrophotometry, as described previously^74^. About 0.5 g of leaf tissue (10 leaves from 3 trees of each replicate) was homogenized in a solution containing 10% trichloroacetic acid (TCA) and centrifuged at 10,000*g* for 10 min to determine MDA. The supernatant aliquot was then treated with 5 mL of 20% TCA containing 0.5% thiobarbituric acid (TBA). The supernatant was allowed to incubate for 60 min at 95 °C before being chilled in an ice bath and centrifuged at 10,000*g* for 10 min. The supernatant absorbance was read at two wavelengths of 532 and 600 nm. After subtracting the non-specific absorbance at 600 nm, the MDA content was defined using an extinction coefficient of 155 mM^−1^ cm^−1^ and expressed as nmol g^−1^ FW.

#### Chlorophylls and total carotenoids

The total carotenoids and chlorophylls were determined as described previously^75^. Briefly, 0.5 g of plant tissue (10 leaves from 3 trees of each replicate) was accurately weighed and homogenized in a tissue homogenizer with 10 mL of 80% acetone. At 4 °C, the homogenized sample mixture was centrifuged for 15 min at 10,000 rpm. The supernatant was separated and examined in a spectrophotometer for the presence of Chlorophyll-a, Chlorophyll-b, and carotenoids. Chlorophyll a, chlorophyll b, total chlorophyll and total carotenoids were determined using the following equations and expressed as milligrams per gram of FW.

$$\begin{gathered} {\text{Chlorophyll a}} = {12}.{\text{25 A}}_{{{663}.{2}}} - {\text{279 A}}_{{{646}.{8}}} \hfill \\ {\text{Chlorophyll b}} = {21}.{\text{5 A}}_{{{646}.{8}}} {-}{ 5}.{\text{1 A}}_{{{663}.{2}}} \hfill \\ {\text{Total chlorophylls}} = {\text{chlorophyll a}} + {\text{chlorophyll b}} \hfill \\ {\text{Total carotenoids}} = \left( {{1}000\,{\text{A}}_{{{47}0}} {-}{ 1}.{\text{82 Chlorophyll a}} - {85}.0{\text{2 Chlorophyll b}}} \right)/{198} \hfill \\ \end{gathered}$$

#### Measurements of quantitative and qualitative attributes of fruits

At the commercial harvest stage in mid-November 2021, all fruits per mutant clone were counted and weighed to quantify total production in terms of the number and yield of ripe and unripe fruits in each tree. In addition, the number of cracked, sunburn, and worm-eaten fruits were counted. Ripe fruits were used for measurements of quality attributes. Three fruits from each tree in each replication were taken randomly for measurements of quality attributes. The fruits were promptly sent to the laboratory for analysis of quality parameters such as fruit volume, diameter, length, total soluble solids (TSS), and titratable acidity (TA). A digital caliper was used to measure the diameter and length of the fruit. Liquid displacement was used to determine the volume of the fruit. The concentration of soluble solids in fruit juice was determined using a temperature-compensated hand refractometer, and titratable acidity was determined in juice by titrating with 0.1 N sodium hydroxide and phenolphthalein as the indicator, with the results expressed as a percentage of citric acid^76^. Citric acid is the main acid responsible for titrable acidity in pomegranate arils^77^.

### Statistical analysis

The analysis of variance was performed on all data using SAS's General Linear Models (GLM) function. The LSD test was used to compare means with a p value ≤ 0.05. In the mutant clones of 'Malase Saveh' pomegranates, Pearson correlation analysis was used to detect connections between several physiological and biochemical features of leaves and some fruit quantitative and qualitative parameters.

### Ethical approval

We confirm that all the experimental research and field studies on plants (either cultivated or wild), including the collection of plant material, complied with relevant institutional, national, and international guidelines and legislation. All of the material is owned by the authors and/or no permissions are required.
